# Supplementary material for: Intermittent cluster dynamics and temporal fractional diffusion in a bulk metallic glass
Source: Nat Commun. 2024 Aug 3;15:6595. doi: 10.1038/s41467-024-50758-3 (PMC11298002; doi:10.1038/s41467-024-50758-3)
Supplement: Supplementary file 1 — Supplementary Information [file 41467_2024_50758_MOESM1_ESM.pdf]

Supplementary Information

Intermittent cluster dynamics and temporal fractional diffusion in a bulk metallic glass

Birte Riechers[1], Amlan Das[2,3], Eric Dufresne[4], Peter M. Derlet*[5], and Robert Maaß*[1,2,6]

[1]Federal Institute of Materials Research and Testing (BAM), Unter den Eichen 87, 12205 Berlin, Germany
[2]Department of Materials Science and Engineering, University of Illinois at Urbana-Champaign, Urbana, IL 61801, USA
[3]Cornell High Energy Synchrotron Source, Cornell University, 161 Synchrotron Drive, Ithaca, New York 14850, USA
[4]Advanced Photon Source, Argonne National Laboratory, 9700 South Cass Avenue, Argonne, IL 60439, USA
[5]Condensed Matter Theory Group, Paul-Scherrer-Institute, CH-5232 Villingen PSI, Switzerland
[6]Department of Materials Engineering, Technical University of Munich, 85748 Garchingen, Germany

X-ray diffractometry

The structural state of the as-cast material was investigated by regular laboratory x-ray diffraction using a Bruker D8 Advance diffractometer equipped with Cu-radiation and a Dectris Eiger2 single-photon detector. Similarly, the initial state of the material was also investigated at the synchrotron prior to the XPCS experiment. Both x-ray patterns shown in Fig. 1 indicate an x-ray

*peter.derlet@psi.ch
*robert.maass@bam.de

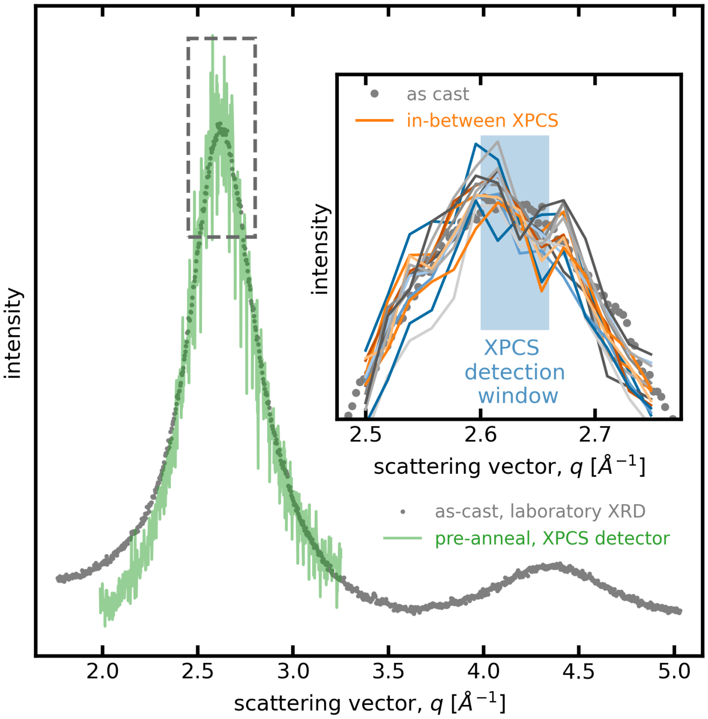

Supplementary Figure 1: **Comparison of x-ray diffraction data.** X-ray diffractogram measured with a conventional X-ray diffractometer on the as-cast material plotted as a function of scattering vector (points), together with diffractograms measured at the beamline on the XPCS-detector (lines) before (main panel) and during (inset) the annealing procedure.

amorphous material prior to the XPCS annealing experiment. To further track the structural stability of the glass throughout the XPCS annealing experiment, a θ-2θ-scan was performed every 8000 speckle patterns over a wider q-range than covered by the speckle patterns themselves (inset in Fig. 1). The duration of such an intermediate θ-2θ-scan amounts to about $180\,\mathrm{s}$, which is significantly shorter than the averaging window for $g_2(t, t_a)$ in the TTCF. The corresponding gaps in the collection of the XPCS-data are thus not visible in the overall TTCF in Fig. 2 and do not affect the analysis of the dynamics in Fig. 3 and Fig. 5 in the main manuscript.

Temperature evolution and thermal stability

The time-evolution of the set-point temperature and the measured sample temperature are presented in Fig. 2. The inset shows the temperature data for to the entire range of the XPCS-experiment, during which the temperature set-point is at $395.00\,°\mathrm{C}$. The mean sample temperature is $0.5\,\mathrm{mK}$ above the set-point temperature, the standard deviation amounts to $2.3\,\mathrm{mK}$. Orange data points represent the sample temperature averaged over 400 frames, directly corre-

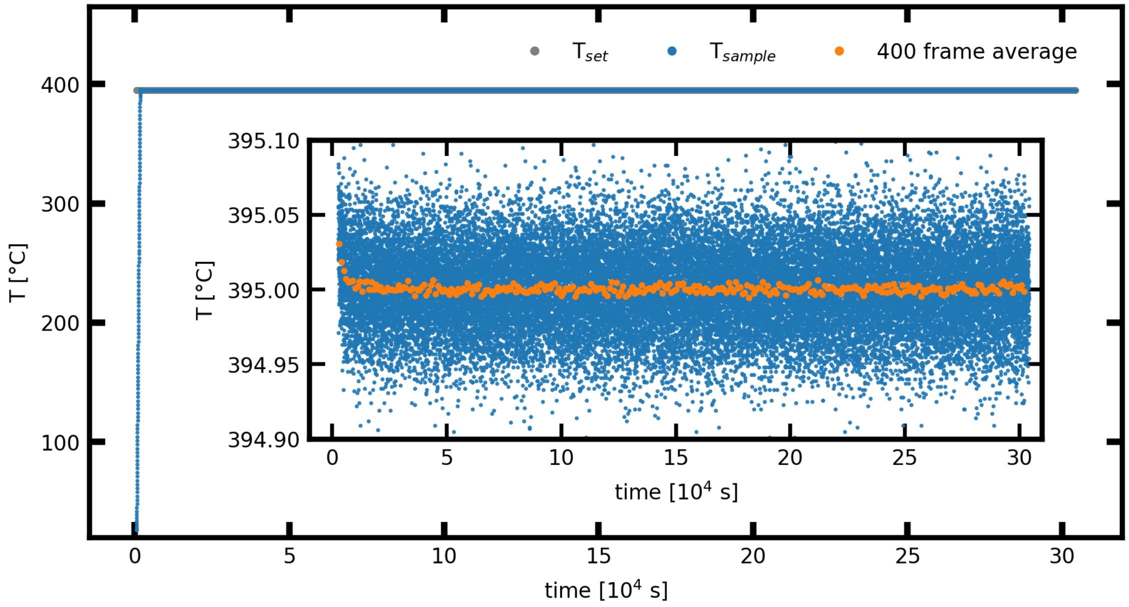

Supplementary Figure 2: **Temperature protocol and accuracy.** Set-point temperature and measured sample temperature as a function of time. The inset shows a magnification of the entire range of the XPCS-experiment with the temperature set-point at $395.00\,^{\circ}\mathrm{C}$, for which the raw and averaged (over 400 frames) sample temperature is displayed.

sponding to the averaging window applied in the analysis of structural dynamics. The initial five data points of the averaged data set illustrate that the sample is very close to the set-point temperature with a maximum deviation of less than $100\,\mathrm{mK}$.

Beam current and scattered intensity

During the experiments, the synchrotron beam current was decreasing with time until a current top-up was conducted. As a function of time, this leads to a saw tooth-like evolution of the beam current and also of the scattering intensity. To demonstrate that beam top-ups are generally not correlated with the discussed fluctuations and features in the short-time plateau and the dynamics of the material, Fig. 3 shows the beam current, I_b (panel a), the scattered intensity recorded on the detector, I_0 (panel b), the short-time plateau, $g_2(0, t_a)$ (panel c), and the decorrelation time, $< t >$ (panel c), as functions of time. As a guide to the eye, point-to-point fluctuations of $g_2(0, t_a)$ and $< t >$ are plotted in the panel d, where high fluctuations would be expected at beam top-ups if these had a strong effect on the intensity-distribution in the recorded speckle patterns. Since fluctuations at beam top-ups do generally not exceed the level of fluctuations at other experimental times, the effect of beam top-ups on the evaluated quantities is found to be negligible. An exception are the last two top-ups where somewhat larger point-to-point fluctuations arise. This does, however, not affect any of the made conclusions. The same applies to measurement disruptions, when intermediate θ-2θ-scans are measured, as well as to the intermittent aging event.

Treatment of decorrelation traces in preparation to fitting procedures

In the following, the text refers to ”decorelation traces” which are hereby defined as the data reflecting decorrelation along a horizontal line in the TTCF at full time-resolution, starting from the TTCF's diagonal, thus corresponding to $g_2(t, t_a) = \langle C(t, t_a) \rangle_{\Delta t_a}$ with Δt_a =2.5 s. The initial value of each decorrelation trace is determined during data processing at the beamline as the average of the contrast values of the four pixels located directly next to the diagonal in the TTCF, that is above, right to, below, and left to the pixel on the diagonal itself. This value is fluctuating over time, reflecting the degree of decorrelation that occurred during the 2.5 s between the first two speckle patterns of a given decorrelation trace. In Fig. 3 of the manuscript, the depicted $g_2(0, t_a)$ is the average over 400 consecutive decorrelation traces (corresponding to a time window of 1000 s) and is displayed together with the resulting standard deviation. This data set is again plotted in Fig. 4. With increasing annealing time, sufficient transport beyond confined atomic positions (an atom does permanently change postion) leads to a reduction in the correlation that in the long-time limit reaches maximum decorrelation and a final plateau. During data analysis and fitting, the final plateau, termed as $g_2(\infty, t_a)$ is assumed to be at a constant value. Figure 4 summarizes both the short- and long-time limit of g_2, where the mean and standard deviation for the last 100 data points of $g_2(t, t_a)$ are plotted. During the initial 20 000 s and the final 40 000 s, the contributing decorrelation traces are clearly too short and the final data points of $g_2(\infty, t_a)$ are still within the decorrelation flank. Between 20 000 s and

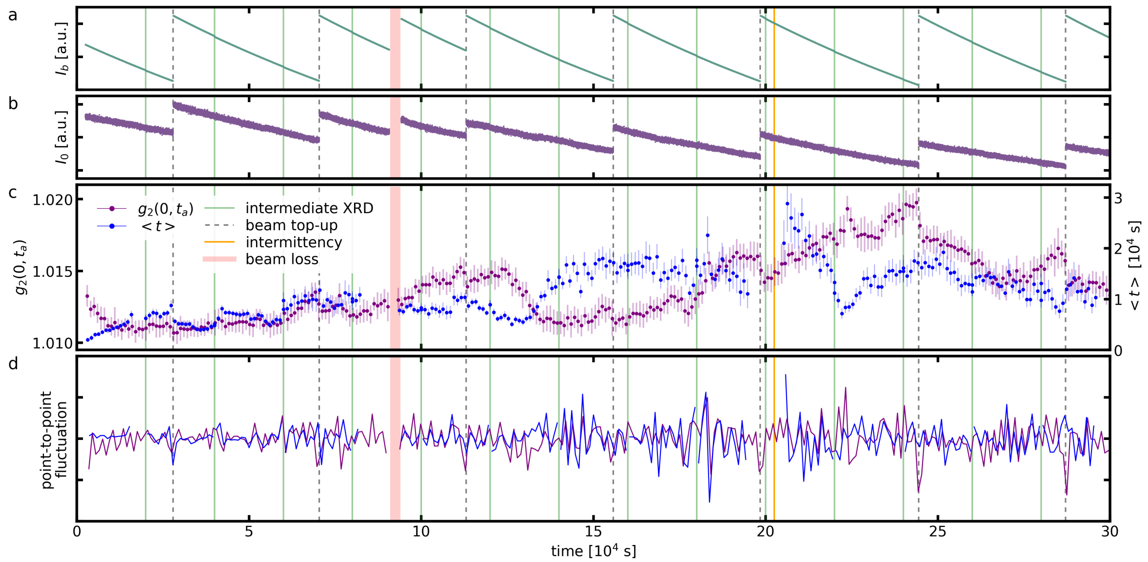

Supplementary Figure 3: **Details of point-to-point fluctuations in decorrelation behavior.** Beam current, I_b, and scattered intensity on the detector, I_0, plotted as functions of time in panels a and b. Panel c shows the evolution of short-time plateau, $g_2(0, t_a)$, and decorrelation time-scale, $< t >$, and their respective standard deviations for comparison, together with their corresponding point-to-point fluctuations in panel d. Vertical lines correspond to intermediate measurements of x-ray patterns to verify the amorphous structure of the glass, occasions of beam top-ups, periods of beam losses, and the occurence of the intermittent event.

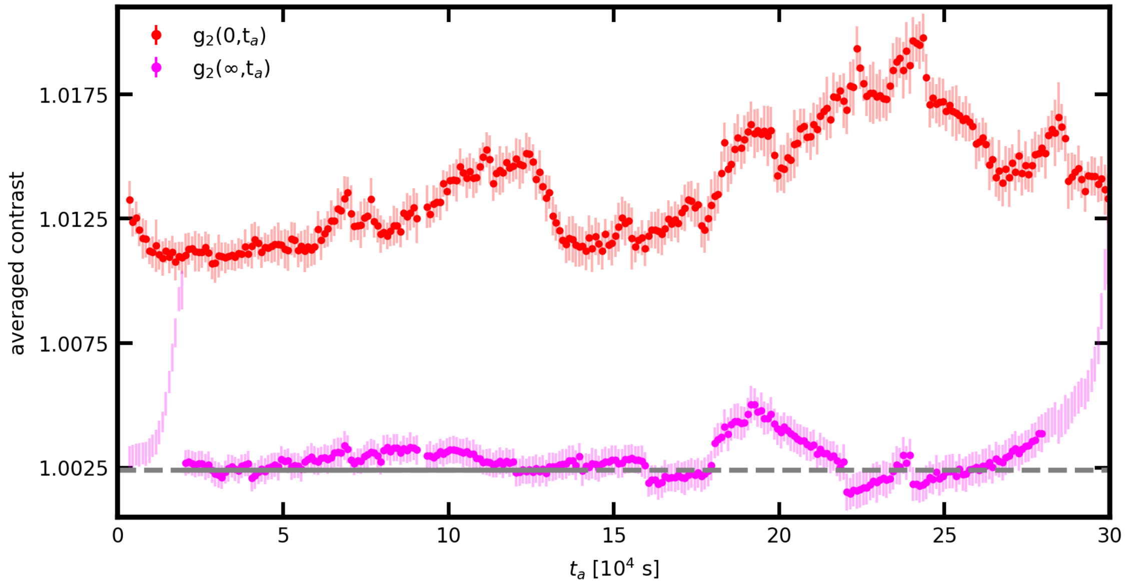

Supplementary Figure 4: **Evolution of short- and long-term plateau.** Initial plateau, $g_2(0, t_a)$, and final plateau, $g_2(\infty, t_a)$, and their respective standard deviations together with the global plateau (grey horizontal line) used to normalize $g_2(t, t_a)$ prior to fitting of the dynamics.

$250\,000\,\mathrm{s}$, the plateau value is, with the exception for waiting times around the intermittent event, steady and indicated with a grey horizontal line at 1.0024.

Fitted pseudo one-time correlation functions, parameter evolution and accuracy of fits

In order to quantify the dynamics throughout the long-term anneal, their evolution is fitted with suitable fitting functions. The conventional approach is represented by fitting with a stretched exponential, the so-called Kohlrausch-Williams-Watts function of the form $f_{\mathrm{KWW}} = \exp\left(-2(t/\tau)^{\beta}\right)$. Assuming a scenario of fractional diffusion, the data is also fitted by the Mittag-Leffler function with the functional form $f_{\mathrm{ML}} = \mathrm{E}_{\beta'}[-q^2 K t^{\beta'}]$. In both cases, a set of 400 consecutive decorrelation traces is averaged and binned to a data point density of 5 data points per decade and subsequently normalized by the individual initial plateau value and the global final plateau. The normalized and binned data, $R(t, t_a)$, are presented in Fig. 5 together with the corresponding fits to the KWW- and ML-functions. The immediate output from the two fitting functions are the time-scale, τ, and the shape exponent, β, which are plotted as a function of annealing time in Fig. 6 together with their standard error based on the covariance matrix deduced during the fitting procedure. From these quantities the first moment and the propagated error of the time-scales are determined. The analysis confirms that the exponents

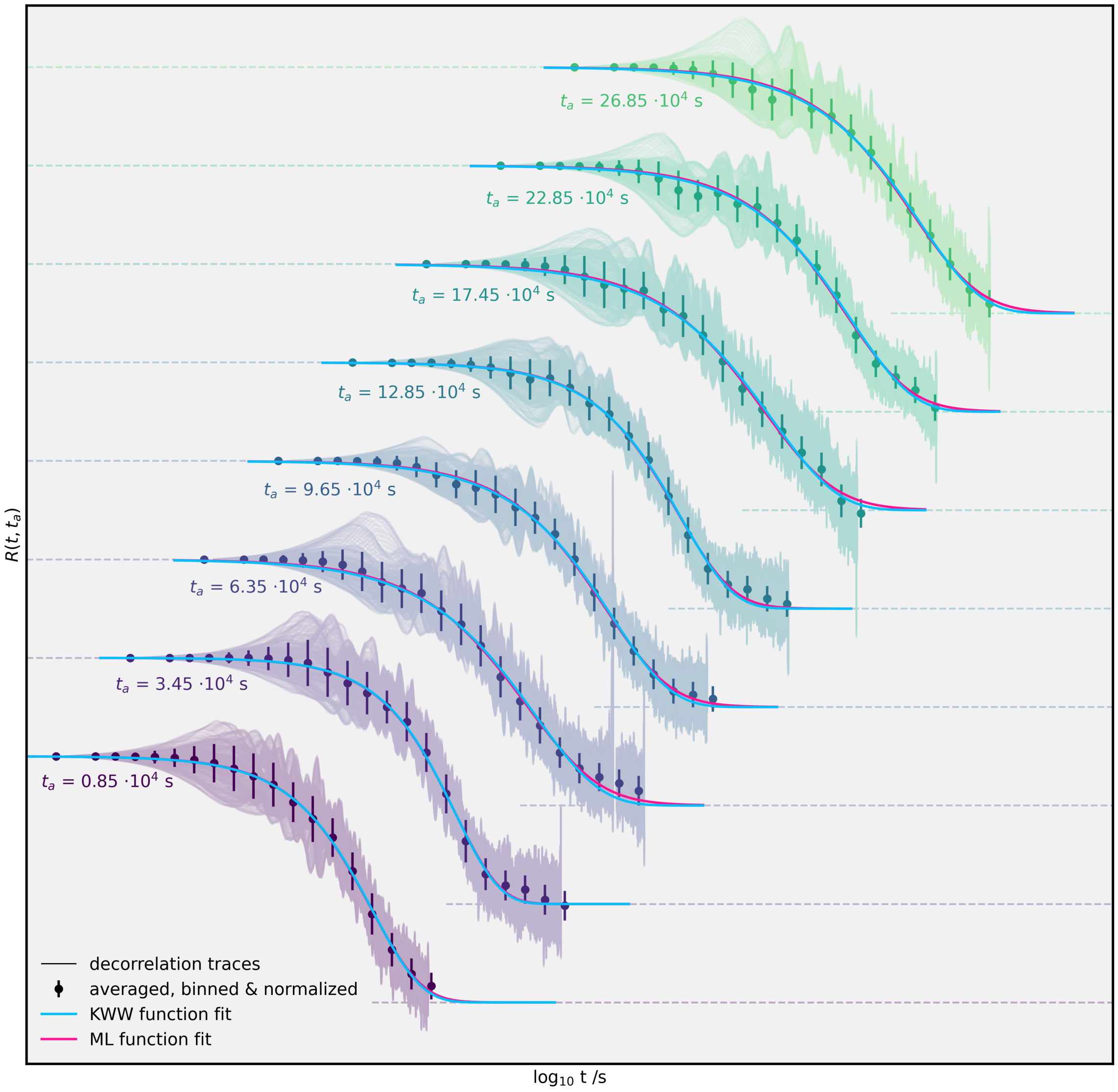

Supplementary Figure 5: **Selection of normalized pseudo one-time correlation functions.** Normalized pseudo one-time correlation functions, $R(t, t_a)$, and their respective standard deviations extracted at annealing times throughout the experiment as a function of time. The thin, colored lines correspond to decorrelation traces at full temporal resolution. The fitting to Kohlrausch-Williams-Watts and Mittag-Leffler functions as indicated by blue and magenta lines is based on $R(t, t_a)$.

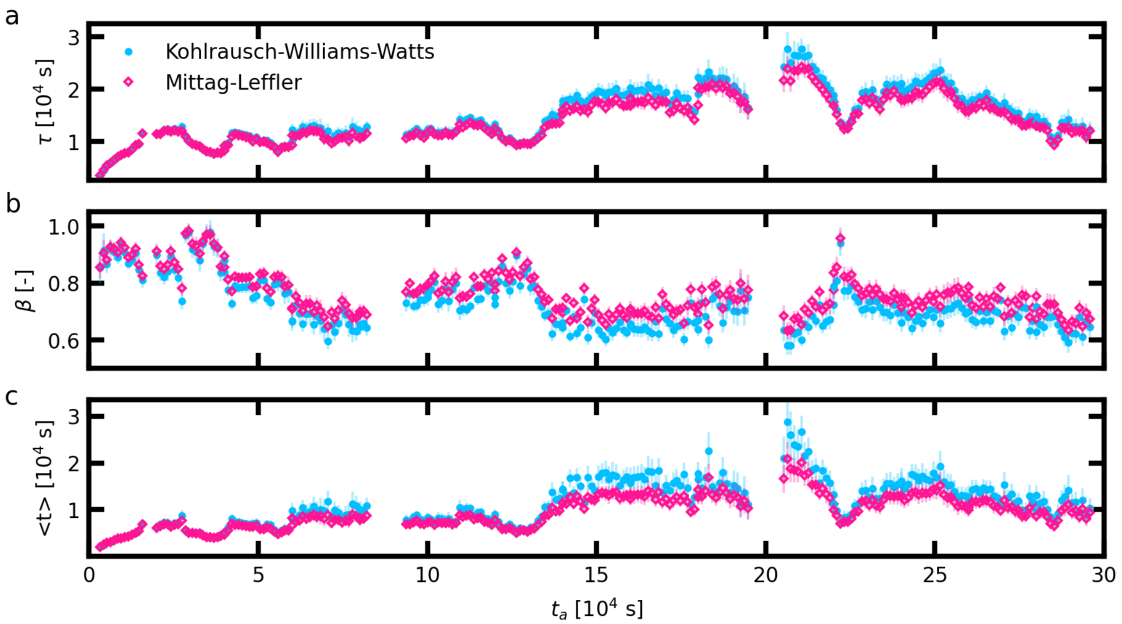

Supplementary Figure 6: **Comparison of fitting results.** Comparison of Kohlrausch-Williams-Watts (KWW) and Mittag-Leffler (ML) fitting results. a) time-scale τ, b) shape exponent β, and c) first moment in time, $< t >$, as functions of annealing time, t_a, each plotted together with the respective standard deviation.

resulting from the ML-fitting are comparable to those deduced by KWW-fitting. While these data correspond to fits of $g_2(t, t_a)$ with $\Delta t_a = 1000\,\mathrm{s}$, the representation in Fig. 5 of the main manuscript assumes that the decorrelation behaves as a true one-time correlation function $g_2(t)$ (i.e., with Δt_a equal to the complete measurement duration), where the fitting parameters of the more suitable Mittag-Leffler function are $\tau = 13\,963\,\mathrm{s}$ and $\beta' = 0.73$. The KWW-function yields the fitting parameters $\tau = 15\,657\,\mathrm{s}$ and $\beta' = 0.67$ for that data set. Note that both sets of fitting parameters are close to the fitted parameters for $g_2(t, t_a)$ with $\Delta t_a = 1000\,\mathrm{s}$ as shown in Fig. 6.

Stability aspects regarding beam and sample

The effect of x-ray flux on glassy dynamics needs to be critically assessed and has been part of earlier XPCS work. For example, Ruta and co-workers give important insights into this topic, such as a study of x-ray beam effects on oxide and metallic glass dynamics [1]. In fact, therein it is demonstrated that a $Cu_{65}Zr_{27.5}Al_{7.5}$ metallic glass is not affected in its dynamics when exposed to an x-ray beam of $1 \times 10^{11}\,\mathrm{ph\,s^{-1}}$. In contrast to the metallic glass, a beam of

$1 \times 10^{11}\,\mathrm{ph\,s^{-1}}$ flux induces more than a ten-fold acceleration in dynamics for a silicon oxide glass. Since the here presented alloy is of similar composition as in the paper by Ruta et al., we assume comparable properties of thermal transport. Furthermore, the flux in the work by Ruta et al. is one order of magnitude larger than the flux in the here presented measurement, underlining flux-independent dynamics in our studied metallic glass.

Further support is given by a study on the influence of high x-ray flux on the shape of the decorrelation function by Gabriel et al. [2]. In this earlier work, material transport due to flow and the exchange of particles beyond the borders of the scattering volume are considered as a result of a beam-sample interaction. Both situations strictly result in decorrelation dynamics that show compressed exponential behavior. The analysis of the dynamics on the here presented data set yields shape exponents that are entirely below unity and therefore reflect stretched decorrelation behavior, as demonstrated in Fig. 6. Based on these insights, we conclude that mechanisms as a consequence of a beam-sample interaction are not dominating the here studied material's dynamics response.

Simulation details

In the present work, atomistic simulations were performed with the LAMMPS software [3] using a binary Wahnström Lennard-Jones pair potential [4] with a one-to-one concentration of small-to-large particles. The LJ potential is chosen because of its fast evaluation, and the Wahnström parameterization because it is known to well describe the bond frustration due to atomic size differences in real binary alloy glasses such as CuZr. Whilst the forces in material specific metallic systems are usually performed via fitted embedded atom potentials, to correctly describe the unsaturated metallic bond, these many-body potentials effectively become pair-potential-like under bulk conditions free from localized free volume such as vacancies and internal surfaces. The glass sample was obtained using an NVT/NPT linear quenching protocol starting from a high-temperature equilibrium liquid phase, defining a (fictive) glass temperature T_0 corresponding to a temperature at which the system froze at the time-scale comparable to the inverse quench rate. At 0.95 T_0, fixed zero-pressure isothermals were performed on a time-scale of $2\,\mu\mathrm{s}$ to further relax the sample, after which the system's temperature was reduced to 0.8 T_0 via an additional linear temperature quench. Fixed zero-pressure isothermal simulations were then performed for $20\,\mu\mathrm{s}$ and used to investigate the transport, structural evolution and decorrelation. More details are given in [5] which considers a similar data-set under both non-zero and zero load.

Supplementary References

[1] Ruta, B. *et al.* Hard x-rays as pump and probe of atomic motion in oxide glasses. *Scientific Reports* **7**, 3962 (2017).

[2] Gabriel, J., Blochowicz, T. & Stühn, B. Compressed exponential decays in correlation experiments: The influence of temperature gradients and convection. *The Journal of Chemical Physics* **142**, 104902 (2015).

[3] Thompson, A. P. *et al.* Lammps - a flexible simulation tool for particle-based materials modeling at the atomic, meso, and continuum scales. *Computer Physics Communications* **271**, 108171 (2022).

[4] Wahnström, G. Molecular-dynamics study of a supercooled two-component lennard-jones system. *Physical Review A* **44**, 3752–3764 (1991).

[5] Derlet, P. M. & Maass, R. Micro-plasticity in a fragile model binary glass. *Acta Materialia* **209**, 116771 (2021).
